# Massive Right Breast Hematoma

**DOI:** 10.5811/cpcem.2018.8.39493

**Published:** 2018-09-05

**Authors:** Manish Amin, Jason P. Jerome, Phillip Aguìñiga-Navarrete, Laura C. Castro

**Affiliations:** Kern Medical, Department of Emergency Medicine, Bakersfield, California

## CASE PRESENTATION

A 53-year-old female with a history of hypertension, congestive heart failure, and generalized anxiety disorder taking 81 milligrams of aspirin daily presented as a trauma activation following a motor vehicle collision. She was the restrained driver of a vehicle traveling at approximately 45 miles per hour that was rear-ended by another vehicle traveling at unknown speed. Airbags were deployed. The patient was extricated by first responders.

**Image 1:**
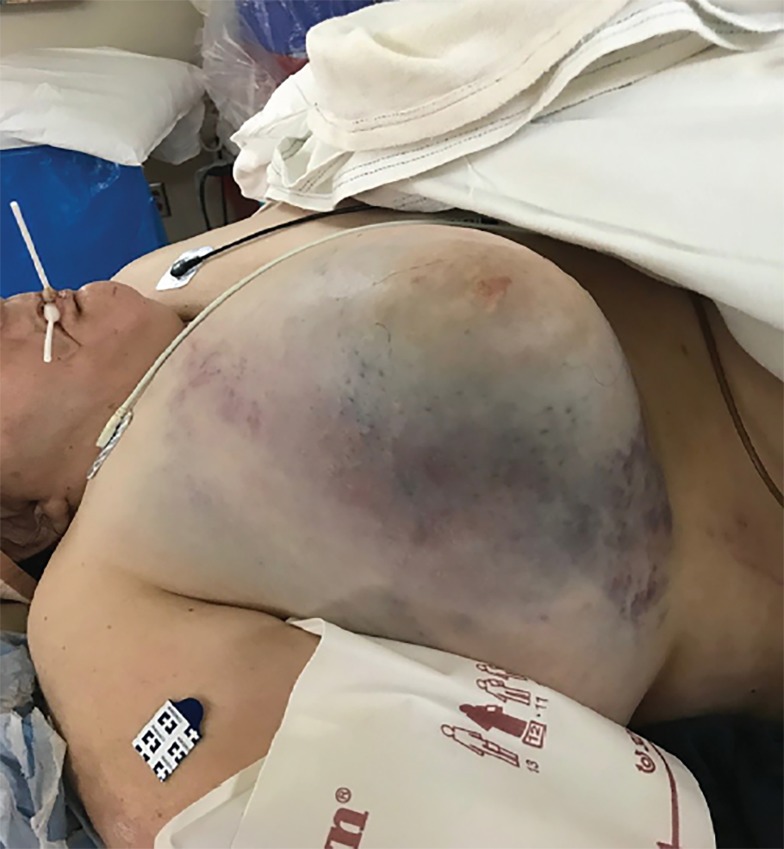
Photograph of patient showing massive right breast hematoma.

Upon presentation to the emergency department she was complaining of severe right breast pain. She was initially tachycardic at 115 beats per minute with a blood pressure of 128/60 millimeters of mercury (mmHg). Her primary survey was intact and her secondary survey was significant for ecchymosis to her right breast, which was swollen, tense and exquisitely tender (Image 1). No further evidence of trauma was noted.

**Image 2:**
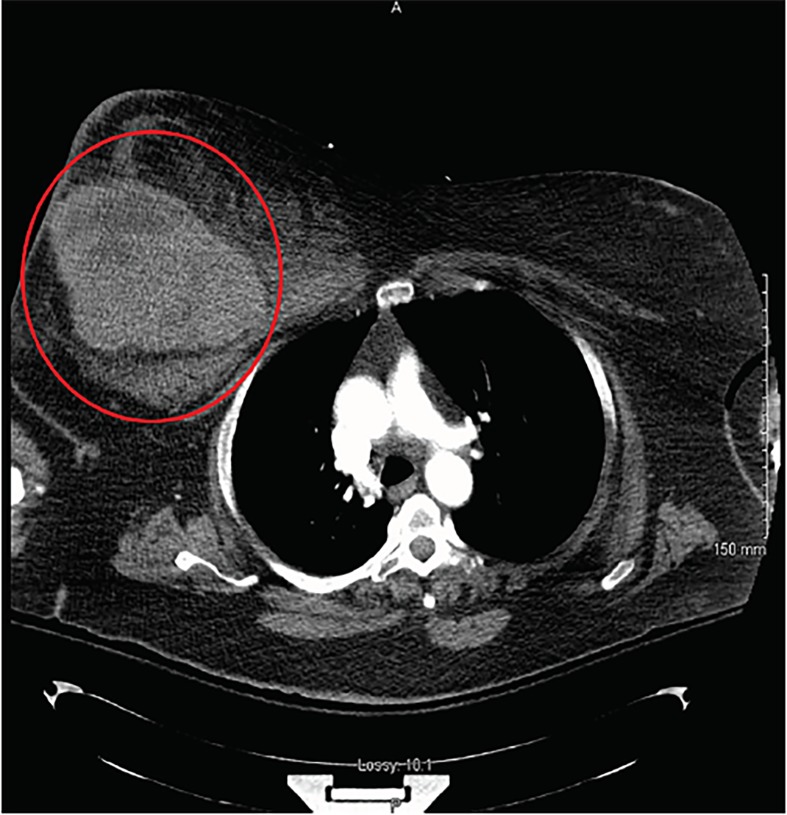
Axial view of a computed tomography of the chest revealing large right breast hematoma (red circle).

After the primary survey her right breast continued to expand and her blood pressure was noted to deteriorate to a recorded low of 99/52 mmHg despite a fluid bolus and blood transfusion. A computed tomography of the chest demonstrated a 10.5 cm × 12.7 cm × 18 cm breast hematoma (Image 2). Remarkably, there was no evidence of other concomitant injuries. Due to her consistently labile blood pressures trauma surgery elected to manage the patient operatively. A 1,500-milliliters hematoma was evacuated, consistent with the patient’s state of class III shock. Origin of the bleeding was determined to be an arterial branch within the pectoralis major. The patient was taking aspirin, causing presumed platelet dysfunction, but her coagulation panel was normal.

## DISCUSSION

Among cases of blunt chest trauma in females, breast hematoma is relatively uncommon, occurring in less than 2%. More than 93.5% are managed expectantly with only 6.5% requiring invasive procedures.^1^ To our knowledge, this is the only reported case of a massive breast hematoma resulting from blunt chest trauma without concomitant injuries demonstrating a state of Class III shock.^2^ A similar computed tomography image has been published.^3^ Nevertheless, the primary difference between this case and the case in the cited image is that the patient in the cited case had concomitant rib fractures and was treated with interventional radiology embolization, whereas the patient in this presentation was treated operatively and had no concomitant injuries. This case illustrates another compartment where hemodynamically-significant bleeding can occur in the setting of trauma.

**CPC-EM Capsule**

What do we already know about this clinical entity?

Breast hematomas are relatively uncommon, occurring in less than 2% of blunt chest trauma. More than 93.5% are managed expectantly, with only 6.5% requiring invasive procedures.

What is the major impact of the image(s)?

To our knowledge, this is the only reported case of an isolated massive breast hematoma resulting from blunt chest trauma demonstrating a state of Class III shock.

How might this improve emergency medicine practice?

It may raise suspicion among emergency providers that hemodynamic compromise may be due to an isolated breast hematoma.

Documented patient informed consent and/or Institutional Review Board approval has been obtained and filed for publication of this case report.
